# Lujan-Fryns syndrome (mental retardation, X-linked, marfanoid habitus)

**DOI:** 10.1186/1750-1172-1-26

**Published:** 2006-07-10

**Authors:** Griet Van Buggenhout, Jean-Pierre Fryns

**Affiliations:** 1 Centre for Human Genetics, University Hospital Leuven, Herestraat 49, B-3000 Leuven, Belgium

## Abstract

The Lujan-Fryns syndrome or X-linked mental retardation with marfanoid habitus syndrome is a syndromal X-linked form of mental retardation, affecting predominantly males. The prevalence is not known for the general population. The syndrome is associated with mild to moderate mental retardation, distinct facial dysmorphism (long narrow face, maxillary hypoplasia, small mandible and prominent forehead), tall marfanoid stature and long slender extremities, and behavioural problems. The genetic defect is not known. The diagnosis is based on the presence of the clinical manifestations. Genetic counselling is according to X-linked recessive inheritance. Prenatal testing is not possible. There is no specific treatment for this condition. Patients need special education and psychological follow-up, and attention should be given to diagnose early psychiatric disorders.

## Disease name/synonyms

Lujan syndrome

Lujan-Fryns syndrome

X-linked mental retardation with marfanoid habitus

## Definition/diagnostic criteria

The Lujan-Fryns syndrome or X-linked mental retardation with marfanoid habitus syndrome (OMIM 309520) is a syndromal X-linked form of mental retardation (mild to moderate mental retardation), associated with tall, marfanoid stature, distinct facial dysmorphism and behavioural problems. The genetic defect is not known.

## Epidemiology

The prevalence in the general population is not known. The Lujan-Fryns syndrome affects predominantly males. In the population of mentally retarded patients and psychiatric patients this syndrome might be more frequent and should be considered in the differential diagnosis of schizophrenia [1].

## Aetiology

The Lujan-Fryns syndrome is a development disorder of genetic origin. However, the cause of this condition is not known. Wittine *et al. *[2] described two related males with ventricular septal defect and progressive aortic root dilatation and suggested that this may implicate a mutation in a structural connective tissue gene.

## Clinical description

**Figure 1:**
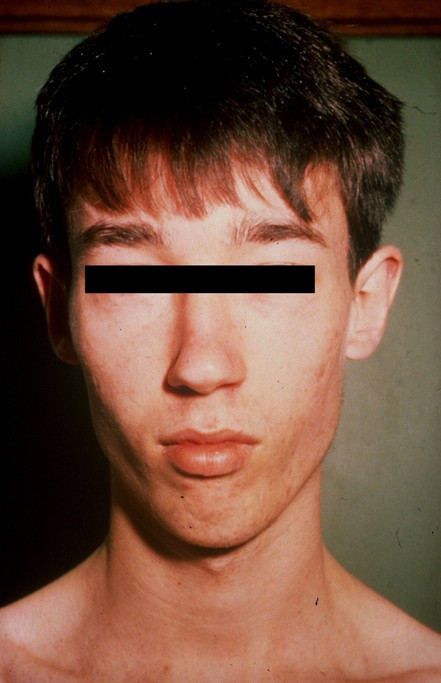
Lujan-Fryns syndrome: note the long narrow face, maxillary hypoplasia, small mandible, long nose, thin upper lip and receding chin.

Patients are mildly to moderately mentally retarded. Craniofacial features include prominent forehead, long narrow face, maxillary hypoplasia, small mandible, long nose with high and narrow nasal bridge, short and deep philtrum, thin upper lip, highly arched palate, receding chin and low-set retroverted normal shaped ears (Figure 1). The marfanoid features include a tall stature, long thin hyper-extensible fingers and toes, but no true arachnodactyly, short halluces, long second toes and sandal gap [4-12]. The marfanoid stature becomes evident after puberty [13]. Adult height is tall, but still in the normal range. There is generalised hypotonia. Joint hyperextensibility and pectus excavatum might be present. Secondary sexual development and testicular size is normal. There is hypernasality, without velopharyngeal incompetence or palatal clefting. Seizures are present in some patients. Structural heart defects have been reported [2,4,14]. Ophthalmologic complications such as lens dislocation are not observed in the Lujan-Fryns syndrome.

Extreme shyness and other behavioural problems were observed in 80% of the cases. Behavioural features include emotional instability, aggression, hyperactivity, shyness and/or autistic behaviour. Psychiatric problems can be present such as psychotic disturbances with hallucinatory visions and sounds, and schizophrenia [1,14-17].

## Diagnostic methods

The diagnosis is based on the presence of the clinical manifestations. There is no specific diagnostic test available. Additional investigations are required to rule out a differential diagnosis and should include a cardiac examination and ultrasound, ophthalmologic examination, chromosomal analysis with special attention for chromosome 5pter (FISH-studies) and 22q11 (FISH-studies), and biochemical analysis of aminoacids in plasma and urine.

## Differential diagnosis

Chromosomal investigation should be done because a chromosomal disorder, such as Klinefelter syndrome (47, XXY) and 47, XYY syndrome, may be present in patients with tall stature. Stahopulu *et al. *[3] described a young man with phenotypical features suggestive of Lujan-Fryns syndrome and autistic spectrum disorder, who has a subtle terminal deletion of the short arm of chromosome 5, suggesting a detailed examination of chromosome 5p to exclude a subtelomeric deletion, by G-banding and FISH-studies.

A 22q11 deletion syndrome (Shprintzen syndrome or Velo-cardio-facial syndrome) should be excluded by FISH-studies in patients who present nasal speech, slender extremities, and psychiatric problems.

The Fragile-X syndrome should be excluded by DNA-analysis of the *FMR1 *gene (molecular investigation the expansion of the CGG repeat in the *FMR1 *gene).

The Marfan syndrome, an autosomal dominant condition, can be excluded by clinical and cardiologic examination, including inspection of the shape of the thorax (pectus excavatum), the presence of features of a generalised connective tissue disorder such as skin striae and scoliosis, and cardiac ultrasound to exclude aortic aneurysm or dissection, mitral and aortic regurgitation and the aortic root diameter should be measured. An ophthalmologic examination should be performed, with slit lamp examination of the eyes to exclude ectopia lentis, myopia or retinal detachment. The condition is caused by mutations of the fibrillin-1 gene (*FBN1*).

Homocystinuria can be excluded by biochemical analysis of aminoacids in plasma and urine, in patients with a tall stature, chest wall deformity and lensdislocation.

## Genetic counselling

Genetic counselling should inform patients of X-linked recessive inheritance. In case of a sporadic patient, there is a recurrence risk of 25% for the following pregnancy. Two affected females have been reported, and the condition could be inherited as an X-linked semi-dominant condition [10,18].

## Antenatal diagnosis

There is no specific prenatal test available for this condition.

## Management including treatment

There is no specific treatment for this condition. Attention should be given to prevent severe scoliosis and progressive orthopaedic problems. Patients with cardiac problems or epileptic seizures should be examined on a regular basis. Patients need special education and psychological follow-up with comprehensive neuropsychological evaluation. Special attention should be given to prevent aggressive outbursts and to diagnose early psychiatric disorders, such as psychosis.

## Prognosis

Most reports describe patients at adolescent and young adult age. Special attention should be given to comorbidity and behavioural problems.

## Unresolved questions

Thus far, the genetic defect and the mechanism is unknown. The condition in female carriers is not known. In one female carrier marfanoid habitus and high pitched nasal speech was seen.
